# Influence of perceived social support and other factors on treatment adherence among adults living with chronic non-communicable diseases in the Ho Municipality of Ghana: A health facility-based cross-sectional study

**DOI:** 10.1371/journal.pone.0308402

**Published:** 2024-09-06

**Authors:** William Kwame Witts, Hubert Amu, Frank Oppong Kwafo, Nathaniel Awentiirin Angaag, Luchuo Engelbert Bain

**Affiliations:** 1 Department of Epidemiology and Biostatistics, Fred N. Binka School of Public Health, University of Health and Allied Sciences, Hohoe, Ghana; 2 Department of Population and Behavioural Sciences, Fred N. Binka School of Public Health, University of Health and Allied Sciences, Hohoe, Ghana; 3 Department of Medical Imaging, School of Allied Health Sciences, University of Health and Allied Sciences, Ho, Ghana; 4 Department of Psychology, Faculty of Humanities, University of Johannesburg, Johannesburg, South Africa; University of Ghana College of Humanities, GHANA

## Abstract

**Background:**

In Sub-Saharan Africa (SSA), there is a noticeable shift from infectious diseases to chronic non-communicable diseases (CNCDs) based on recent studies. However, other studies suggest that social support can significantly improve self-care, increase knowledge of disease symptoms, and ultimately increase overall well-being in patients with CNCDs. In this study, we investigated the influence of perceived social support on treatment adherence among adults living with CNCDs in the Ho Municipality.

**Methods:**

This was a health facility-based cross-sectional study among 432 adults living with cancer, diabetes, chronic kidney disease (CKD), stroke, and hypertension in the Ho Municipality of the Volta Region, Ghana. We adopted the Multi-dimensional Scale of Perceived Social Support (MSPSS), Medication Adherence Rating Scale and independent items to collect data. Logistic regression models were used to analyze the data with STATA v17.0 at 95% Confidence Intervals with statistical significance set at p<0.05.

**Results:**

Majority of the participants (62%) reported high levels of perceived social support. While friends were the main source of support (69.4%), significant others provided the least support (45.4%). Among the dimensions of treatment adherence, participants demonstrated the highest adherence to reviews/check-ups (98.8%), while medication adherence had the highest level of non-adherence (38%). We did not find a significant association between perceived social support and overall treatment adherence, except for individuals with low perceived social support from friends (aOR = 8.58, 95% CI = 4.21,17.52), who were more likely to exhibit high adherence to behavioural and lifestyle recommendations.

**Conclusion:**

While the majority of respondents reported high perceived social support, there was no significant link between social support and overall treatment adherence. However, individuals with low support from friends showed a notably increased adherence to behavioural and lifestyle recommendations. This underscores the nuanced impact of social support on specific aspects of adherence, highlighting the need for targeted interventions tailored to individual support networks.

## Introduction

Chronic non-communicable diseases (CNCDs) are a major global public health challenge, responsible for 74% (41 million) of all deaths worldwide [1]. Most of these CNCDs are attributed to genetic, physiological, behavioural, or environmental factors or a combination of them [2]. The impact of CNCDs on individuals, families, and societies is enormous, and they represent a major barrier to the achievement of the sustainable development goals (SDGs) by the year 2030; specifically, the SDG target 3.4 which seeks to reduce premature mortality from non-communicable diseases by one third [3]. The burden of CNCDs is likely to increase in the coming years, with factors such as ageing populations, changing lifestyles, and urbanization contributing to the rise in prevalence [4].

Sub-Saharan Africa, where a significant proportion of the population falls within the lowest socio-economic category, bears a disproportionate share of the global CNCD burden [5, 6]. It was estimated that disability-adjusted life years (DALYs) attributable to non-communicable diseases in SSA went from 90.6 million in 1990 to 151.3 million in 2017 [7]. This exponential increment in DALYs underscores the burden of CNCDs in SSA. It has been projected that SSA would experience one of the largest increases in mortality due to CNCDs globally and an estimated increase in deaths by 46% of all deaths by the year 2030 if immediate measures are not taken [7, 8]. While infectious diseases have traditionally dominated Sub-Saharan Africa (SSA), studies have shown an increasing prevalence of CNCDs including cancer, diabetes, hypertension, and cardiovascular diseases across SSA countries [7, 9, 10]. In comparison to infectious diseases, there has been inadequate progress in preventing and controlling CNCD-related deaths as managing CNCDs usually requires a great deal of commitment from the individual, family, and society [11].

Ghana, like many other SSA countries, is burdened with a high prevalence of CNCDs which are responsible for around 43% of all deaths in the country [12]. The most common CNCDs in Ghana are cardiovascular diseases (19%), cancers (5%), chronic respiratory diseases (3%), and other CNCDs (13%). Furthermore, Ghana has reported a 21% risk of premature mortality due to CNCDs [12, 13].

The treatment for CNCDs usually includes long-term pharmacotherapy and behaviour modification. However, poor treatment adherence has been acknowledged as a significant barrier to improving patient outcomes [14, 15]. Social support has become a health determinant that has been studied on several occasions to determine its level of influence [16–19]. Some studies have revealed that treatment adherence is positively impacted by adequate and enhanced social support, which improves patients’ quality of life by minimizing social isolation [9, 20, 21]. An investigation into the influence of social support on treatment adherence among diabetic patients revealed a significant association between social support and medication adherence [17]. Similarly, a study which sought to ascertain the role of religious coping and social support on medication adherence and QoL in elderly diabetic patients found that the impact of religiosity on medication adherence and HRQoL occurs through mediators such as religious coping and social support [22].

Although there are some studies [23–25] on the subject, there remains a dearth of research examining the relationship between social support and adherence behaviours, such as medication adherence, lifestyle/behavioural modifications, as well as review adherence among adults living with CNCDs especially in Ghana. Hence, this study sought to provide a comprehensive understanding of the relationship between perceived social support and treatment adherence in CNCD patients in Ghana and the predictors of treatment adherence. The findings could positively inform policy decisions on the management of CNCDs in Ghana and beyond.

We relied on the Strengthening the Reporting of Observational Studies in Epidemiology (STROBE) statement in writing the manuscript **[S1 Checklist]** [26].

## Materials and methods

### Study site

The Ho Teaching Hospital (HTH), situated in the Ho Municipality of the Volta Region, provides both out-patient and in-patient services. The hospital has five clinical departments: internal medicine, surgery, obstetrics and gynaecology, child health, and public health. The HTH’s clinical services are broadly divided into two categories: general and specialist clinical services. General services include general surgery, urology, paediatrics, orthopaedics, etc., whereas specialist services are delivered by disease-/condition-specific clinics such as the diabetes clinic, fertility clinic, physiotherapy, anti-retroviral therapy clinic, and eye clinic [27].

The HTH was selected for this study because it has become one of the top hospitals in the country and receives both primary and referral cases from the entire Volta Region and parts of the neighbouring regions in Ghana and provides access to populations of interest.

### Study design

This study forms part of a larger study which quantitatively explored the psychosocial aspects of treatment adherence among adults living with chronic non-communicable diseases in the Ho Municipality. This was a health facility-based study which utilized a quantitative descriptive cross-sectional design in obtaining data from respondents to determine the influence of respondents’ perceived social support on their treatment adherence behaviour.

### Study population

The target population of this study were adults diagnosed with hypertension, cancer (breast, cervical, prostate, or lung cancers), chronic kidney disease (CKD), stroke and/or diabetes. Adults with any of these CNCDs of interest who visited the hospital’s disease-/condition-specific clinics and chronic disease patients in in-patient care units were included in the study. In contrast, adults with any of the CNCDs of interest who visited the hospital’s disease-/condition-specific clinics but were foreign nationals, severely or terminally ill and unable to effectively communicate were excluded from participating in this study.

### Sample size determination

The minimum sample size to be used was determined using the single proportion population formula by Cochran (1977) [28]: $n=\frac{\left({\text{z}}^{2}\text{p}(1-\text{p})\right.}{{\text{d}}^{2}}$, where; n = sample size, z = selected critical value of desired confidence level, p = estimated proportion of an attribute that is present in the population and d = precision (corresponding to effect size). The sample size was estimated based on 50% (0.5) prevalence estimates of CNCDs in Ghana [29].


$$n=\frac{{\left(1.96\right)}^{2}(0.5)(1-0.5)}{{\left(0.05\right)}^{2}}$$

n = 384.16

n ≈ 385

We adjusted for a 10% non-response rate. Adding the calculated sample size and the anticipated non-response response rate, a minimum sample size of 424 was calculated for this study.

### Sampling technique

A probabilistic sampling method utilizing the simple random sampling technique was adopted in recruiting respondents for the study. This was done by utilizing the various units’/clinics’ attendance registers to aid in getting the lists of clients with the various CNCDs to be under-studied. Individuals who gave informed consent were then asked to pick a piece of paper with random numbers, which were generated using Microsoft Excel. A total of 432 random numbers between 1 and 600 were generated. Individuals who selected a number from the list of random numbers were then enrolled in the study.

### Data collection

Structured interviewer-administered questionnaires were used in collecting data from respondents who met the eligibility criteria. Although data collection was at the hospital, interviews were conducted at secluded locations. The data collection instrument utilized consisted of three sections–respondents’ socio-demographic characteristics, respondents’ PSS and respondents’ adherence to treatment **[S1 Data].** The study adopted the Multi-dimensional Scale for Perceived Social Support (MSPSS) and Medication Adherence Rating Scale (MARS) protocols to measure the PSS and medication adherence of patients with independently developed items measuring both respondents’ behavioural/lifestyle and review adherence. Subsequently, a pilot study was conducted among 50 adults living with CNCD from the Volta Regional Hospital in the Hohoe Municipality (also located within the Volta Region of Ghana). The results of the pilot study were then used to further simplify the questions to avoid ambiguity and to enable the researchers to collect data from participants with low or no proficiency in English. The data collection for this study commenced on the 11^th^ of August 2022 and concluded on the 30^th^ of September 2022.

### Research team

The first, third, and fourth authors–all males and final-year undergraduate students were involved in collecting the data. Prior to the data collection, the three individuals were trained by the second (male, PhD) and fifth (male, PhD) authors.

### Study variables

#### Outcome variables

The secondary outcome variable for this study was the treatment adherence behaviour of respondents. The treatment adherence variable was designed to encompass three distinct dimensions of treatment. These were medication, behavioural/lifestyle and review/check-up adherences.

The medication adherence of respondents was measured using the Medication Adherence Rating Scale (MARS). The MARS [30] is a ten-item yes/no self-report instrument. It was developed from two existing scales, the 30-item Drug Attitudes Inventory (DAI) [31] and the 4-item Medication Adherence Questionnaire (MAQ) [32], to develop a more reliable and valid tool for assessing medication adherence behaviour in psychosis. Total scores range from 0 (low medication adherence) to 10 (high medication adherence). This reflects an understanding that adherence is a continuous variable.

The behavioural/lifestyle and review adherence of respondents were assessed using independent items developed by investigators. Both dimensions of adherence were assessed by asking respondents “*How often do you adhere to these recommendations (behavioural/lifestyle changes)*?” and “*How often do you adhere to your scheduled appointments for your follow-up/check-up/review*?*”* with the former measuring behavioural/lifestyle adherence while the latter measured review adherence”

#### Key explanatory variable

The key explanatory variable for this study was PSS. This was measured using the Multidimensional Scale of Perceived Social Support (MSPSS) [33]. The MSPSS is a 12-item questionnaire to identify an individual’s perceived level of social support with family, friends, and significant others. The MSPSS comprises three subscales with each containing four items. The family, friend and significant other subscales are comprised of items 3, 4, 8, and 11; 6, 7, 9, and 12; and 1, 2, 5, and 10 respectively. Scores for PSS were calculated using the mean scores for the relevant items for each subscale with respondents’ overall PSS calculated using the mean scores of their responses across the three subscales.

#### Other explanatory variables

Age, sex, marital status, education, religion, ethnicity, diagnosed CNCD, diagnosis duration, comorbidity status, specific comorbidities, recommended behavioural/lifestyle changes: physical activity, dietary changes, smoke cessation, and alcohol intake moderation were the explanatory variables used for this study. These variables were selected based on their relevance to the study, as found by other studies. Table 1 is an appendix including an operational definition of study variables **[S1 Table]**.

**Table 1:** Socio-demographic characteristics of respondents.

Characteristics	Frequency [N = 432]	Percentages [%]
Age (In Years) [Mean (SD)]	[58.74(11.31)]	
30–39	38	8.8
40–49	43	9.95
50–59	145	33.6
60+	206	47.7
**Sex**		
Male	205	47.4
Female	227	52.6
**Marital Status**		
Never married	34	7.9
Married	301	69.7
Divorced/Separated	20	4.6
Widowed	77	17.8
**Highest Education Level**		
No formal education	16	3.7
Primary	28	6.5
JHS/JSS/Middle School	100	23.2
SHS/SSS/O-Level	112	25.9
Tertiary	176	40.7
**Religion**		
Christianity	360	83.3
Islam	67	15.5
African Traditional	5	1.2
**Ethnicity**		
Akan	140	32.4
Ewe	235	54.4
Guan	30	6.9
Ga/Dangme	27	6.3
**Diagnosed CNCD**		
Cancer	16	3.7
Chronic Kidney Disease	56	13
Diabetes	52	12
Hypertension	188	43.5
Stroke	120	27.8
**Cancer Type**		
Breast Cancer	6	37.5
Prostate Cancer	9	56.3
Bladder Cancer	1	6.2
**Diagnosis Duration (**Year)		
<1	97	22.5
1–5	176	40.7
6–10	101	23.4
10+	58	13.4
**Living with Comorbidities**		
Yes	167	38.7
No	265	61.3
**Comorbidities**		
Arthritis	4	2.45
Bodily pains	4	2.45
Diabetes	20	12.3
Diabetes &Hypertension	8	4.9
Hypertension	110	67.5
Hernia	1	0.6
Peptic ulcer	4	2.45
Rheumatism	4	2.45
Stroke	8	4.9
**Current behavioural/lifestyle treatment**		
No	4	0.9
Yes	428	99.1
**Recommended behavioural/lifestyle changes**		
**Dietary Changes**		
No	65	15.3
Yes	359	84.7
**Physical Activity**		
No	31	7.3
Yes	393	92.7
**Smoke Cessation**		
No	422	99.5
Yes	2	0.5
**Alcohol Intake Moderation**		
No	354	83.5
Yes	70	16.5
**Adherence to recommendation(s)**		
All the time	117	27.3
Most of the time	245	57.3
Sometimes	54	12.6
Rarely	12	2.80
**Follow-up/check-up/review**		
Weekly	133	31.1
Every two weeks	73	17.1
Monthly	141	33
Every two months	68	16
Every three months	13	2.8
**Adherence to scheduled appointments for follow-up/check-up/review**		
All the time	184	43
Most of the time	239	55.8
Sometimes	0	0
Rarely	5	1.2

### Statistical analyses

Data analyses were done using Stata software version 17.0 (Stata Corporation, College Station, TX, USA). Firstly, means and standard deviations were used to describe continuous variables whereas proportions were used to describe the categorical variables. The PSS of respondents was estimated using the reference provided by Zimet et al. [33] and was further dichotomized using the mean value of their responses to the MSPSS protocol [33]. Where all scores below the mean score were categorized as low PSS and scores above the mean score were categorized as high PSS. Univariable and multivariable logistic regression analyses were further performed to ascertain the association between the outcome variable and explanatory variables. A p-value less than 0.05 was considered statistically significant in this study. Variables that showed significance (p<0.05) in the univariable analysis were included in the multivariable model in which statistical significance was considered at p<0.05

### Ethics considerations and consent to participate

The study was approved by the Research Ethics Committee of the University of Health and Allied Sciences with the reference number: **UHAS-REC A10 (70) 21–22**. Further authorization was obtained from the Research Department of the Ho Teaching Hospital. Consent to participate in the study was also obtained through written informed consent forms from the participants. All methods were carried out according to relevant guidelines and regulations. Access to data collected for this study was limited to study investigators only and kept

## Results

### Socio-demographic characteristics

Table 1 presents the socio-demographic characteristics of the respondents. The mean age was 58.74±11.31. About 48% are over 60 years old. The majority (52.6%) were females, married (69.7%), Christians (83.3%), and Ewes (54.4%). A comparative majority also had a tertiary-level education (40.7%). Amongst the 5 CNCDs of interest in this study, 43.5% of the total study population reported being diagnosed with hypertension. About 41% of the respondents also reported living with their respective CNCDs for a minimum of a year and a maximum of five (5) years while only 38.7% of the study population reported living with at least one comorbidity. Furthermore, 99.1% of the study respondents reported being on behavioural and lifestyle treatment with 84.7%, 92.7%, and 16.5% reporting being recommended dietary changes, physical exercise/activity and alcohol intake cessation respectively.

### Perceived social support

**Fig 1:**
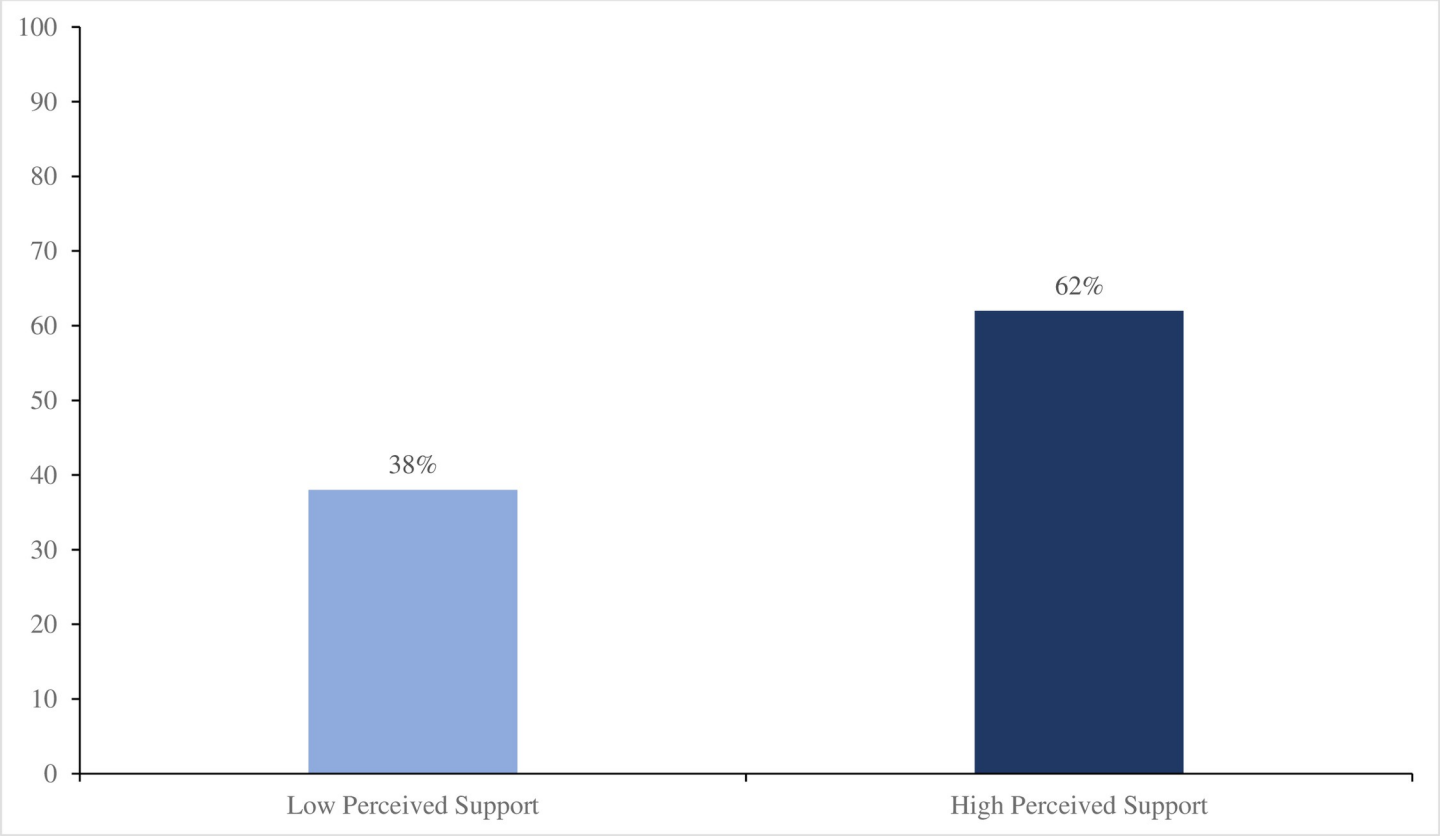
Levels of perceived social support.

Fig 1 shows the perceived social support levels among respondents. The study results found a relatively high level of perceived social support among respondents where 62% of respondents reported high perceived support whereas 38% reported low support.

### Perceived social support subscales

**Fig 2:**
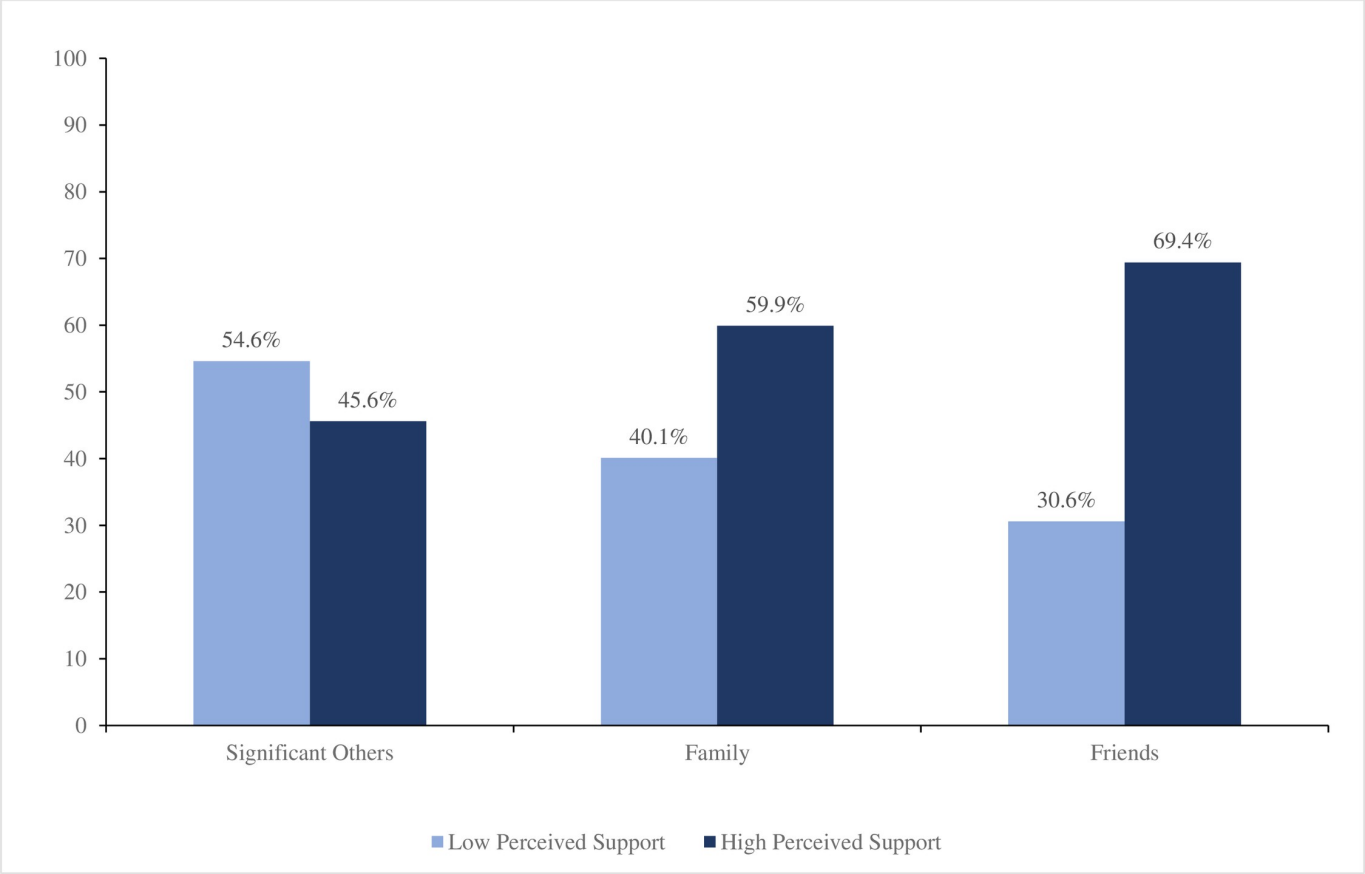
Perceived social support subscale.

Fig 2 presents the findings pertaining to perceived social support levels based on subscales indicating that the respondents received the highest degree of support from their friends. Specifically, the respondents reported a perceived support rate of 69.4% from their friends, which represents the highest level of support across the three subscales of social support. Conversely, the respondents reported the lowest level of support from their significant others, with only 45.4% perceiving support from this source. Notably, family members were reported to provide high levels of support by 59.9% of the respondents.

### Treatment adherence levels

**Fig 3:**
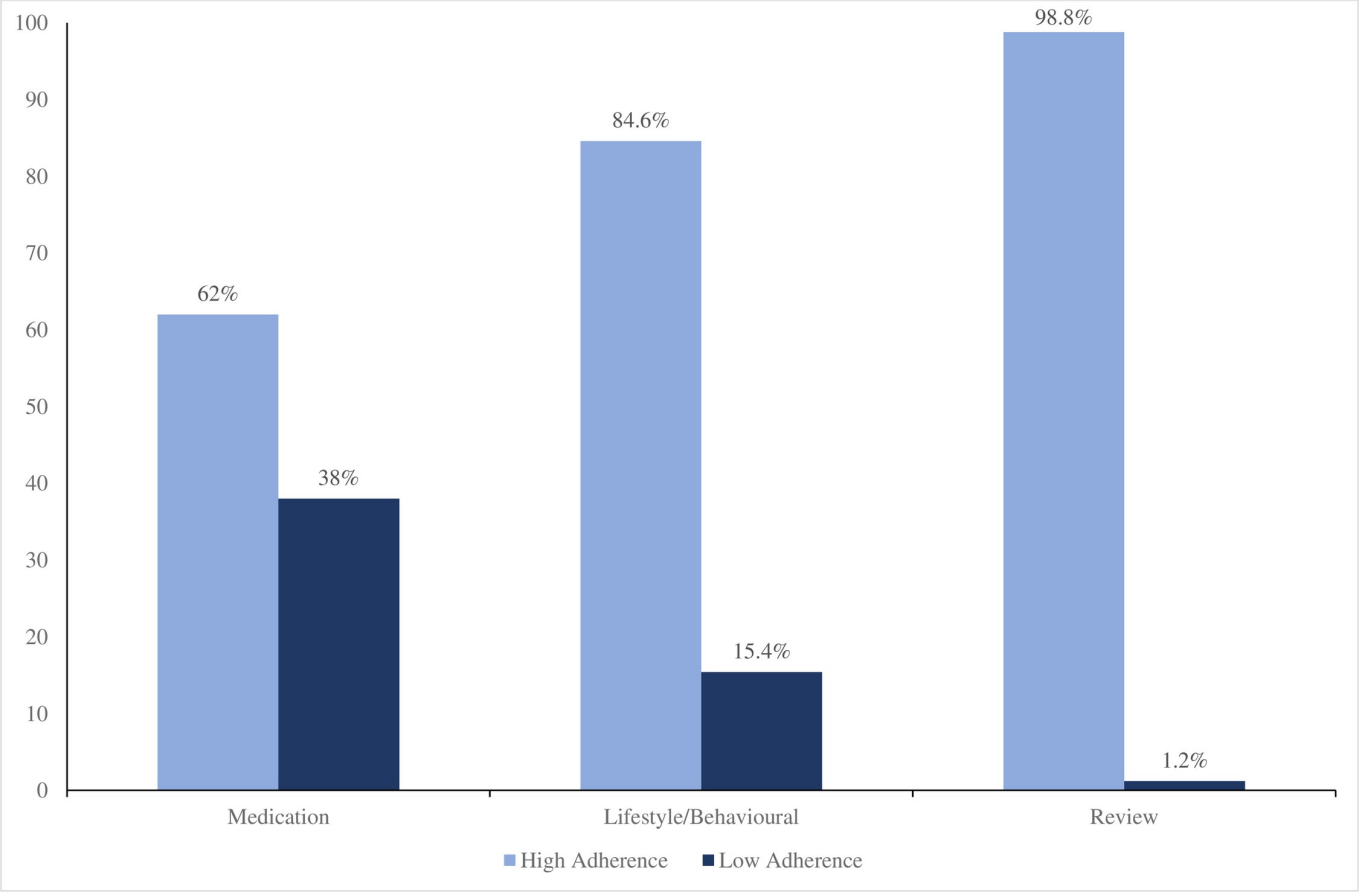
Treatment adherence levels of respondents.

Fig 3 illustrates the levels of adherence of respondents. The study found that respondents reported high levels of adherence across the three dimensions of treatment adherence of interest. The results further revealed that 62%, 84.6% and 98.8% of respondents showed high levels of adherence to their medications, lifestyle/behavioural treatments as well as review schedules, respectively. Respondents reported the highest rate of non-adherence with their medications (38%)

### Predictors of treatment adherence

Table 2 presents the summaries of the logistic regression model run on respondents’ socio-demographic characteristics and their treatment adherence. The results revealed that respondents’ religion, diagnosed CNCDs, ethnicity, diagnosis duration and physical activity recommendation were predictive of medication adherence with respondents of the Islamic (aOR = 2.38, 95% CI = 1.23, 4.60) and African Traditional (aOR = 42.03, 95% CI = 3.18, 555.50) faiths, as well as respondents diagnosed with diabetes (aOR = 7.95, 95% CI = 1.36, 46.59) and hypertension (aOR = 7.48, 95% CI = 1.33, 42.0), being more likely to report high adherence with their medication. On the other hand, respondents of the Ga/Dangme ethnic group (aOR = 0.19, 95% CI = 0.05, 0.70) were less likely than those of the Akan ethnic group whereas those who are Guans (aOR = 3.20, 95% CI = 1.16, 8.82) were more likely to be adherent to their medications than Akans. Also, respondents who had been living with their diagnoses for more than a year and those whose physical activity (aOR = 0.23, 95% CI = 0.87, 5.89) was recommended were less likely to be adherent to their medication. Furthermore, respondents’ lifestyle/behavioural adherence was predicted by their age, level of education, diagnosed CNCD as well as the duration for which they have been living with their diagnoses. Respondents between the ages of 50–59 years, those diagnosed with CKD as well as those living with their diagnoses for more than a year were less likely to be adherent to lifestyle/behavioural recommendations geared to manage their conditions but respondents whose highest level of education is primary school were more likely to be adherent to the lifestyle/behavioural recommendations.

**Table 2:** Predictors of treatment adherence.

Variables	High Medication Adherence		High Lifestyle/Behavioural Adherence		High Review Adherence	
	COR [95% CI] p-value	AOR [95% CI] p-value	COR [95% CI] p-value	AOR [95% CI] p-value	COR [95% CI] p-value	AOR [95% CI] p-value
**Age group**						
30–39 Years	Ref	Ref	Ref	Ref	Ref	
40–49 Years	0.94(0.39, 2.26)	3.81(0)0.990	1.66(0.64, 4.29)	3.43(0.90, 3.06)	0.84(0.09,7.71)	
50–59 Years	0.57(0.28, 1.16)	7.90(0)0.989	0.17(0.06, 0.45) ***	0.13(0.03, 0.70) **	1	
60+ Years	0.42(0.21, 0.85)	9.00(0)0.990	0.53(0.23, 1.19)	0.37(0.09, 1.50)	1	
**Sex**						
Female	Ref		Ref		Ref	
Male	1.09(0.74, 1.61)		0.77(0.45,1.31)		4.42(0.49, 39.86)	
**Marital Status**						
Never married	Ref	Ref	Ref		Ref	
Married	2.33(1.03, 5.34) *	2.13(0)0.988	0.58(0.22, 1.51)		1	
Divorced/Separated	4.87(1.48, 16.11) **	3.53(0)0.988	3.11(0.89, 10.91)		1	
Widowed	0.99(0.38, 2.57)	1.80(0)0.989	1.53(0.55, 4.25)		1	
**Highest Education Level**						
No formal education	Ref		Ref	Ref	Ref	
Primary	0.75(0.22, 2.57)		8.44(3.56, 20.03) ***	101.08(17.06, 598.92) ***	1	
JHS/JSS/Middle School	0.51(0.18, 1.49)		1.88(0.99, 3.58)	1.56(0.62, 3.92)	1	
SHS/SSS/O-Level	0.37(0.13, 1.06)		0.24(0.08, 0.69) **	0.55(0.15, 2.05)	0.39(0.043, 3.51)	
Tertiary	0.83(0.30, 2.32)		1	1	1	
**Religion**						
Christianity	Ref	Ref	Ref		Ref	
Islam	2.21 (1.30, 3.74) **	3.10 (1.66, 5.81) **	0.60 (0.26, 1.38)		0.60 (0.26, 1.38)	
African Traditional	7.61 (0.84, 68.85)	18.72(1.51, 232.58)**	1.28 (0.14, 1.70)		1.28 (0.14, 1.70)	
**Ethnicity**						
Akan	Ref	Ref	Ref	Ref	Ref	Ref
Ewe	0.98 (0.63, 1.51)	0.71 (0.42, 1.21)	2.72 (1.39, 5.34) **	1.09 (0.46, 2.59)	2.72 (1.39, 5.34) **	0.11 (0.011, 0.07)
Guan	2.54 (1.13, 5.69) *	3.94 (1.47, 10.58) *	1.64 (0.49, 5.49)	3.46 (0.75, 16.03)	1.64 (0.49, 5.49)	1
Ga/Dangme	0.71 (0.29, 1.74)	0.30 (0.09, 1.02) **	1.33 (0.35, 5.08)	0.19 (0.04, 1.01)	1.33 (0.35, 5.08)	1
**Diagnosed CNCD**						
Cancer	Ref	Ref	Ref	Ref	Ref	Ref
Chronic Kidney Disease	0.16 (0.49, 0.54) **	0.28 (0.06, 1.35)	0.17 (0.04, 0.73) **	0.09 (0.01, 0.87) *	0.17(0.04–0.73)0.018	1
Diabetes	0.51 (0.1, 1.62)	3.15 (0.61, 16.38) *	0.18 (0.04, 0.8) *	2.42 (0.22, 26.93)	0.18 (0.04, 0.8) *	1
Hypertension	0.32 (0.11, 0.93) *	1.74 (0.37, 8.16) *	0.80 (0.26, 2.41)	4.03 (0.53, 30.52)	0.80 (0.26, 2.41)	0.77 (0.06, 9.45)
Stroke	0.46 (0.16, 1.34)	0.86 (0.20, 3.61)	0.08 (0.02, 0.32) ***	0.12 (0.01, 1.01)	0.08 (0.02, 0.32) ***	1
**Diagnosis Duration**						
<1 Year	Ref	Ref	Ref	Ref	Ref	Ref
1–5 Years	1.09 (0.66, 1.80)	0.83 (0.43, 1.61) **	0.26 (0.12, 0.57) ***	0.03 (0.01, 0.14) ***	1.01 (0.61, 1.67)	1
6–10 Years	0.79 (0.44, 1.39)	0.25 (0.10, 0.64) ***	0.96 (0.48, 1.94)	0.09 (0.02, 0.44) **	0.56 (0.32, 0.99) *	1
10+ Years	0.10 (0.03, 0.29) ***	0.04 (0.01, 0.15) ***	1.48 (0.69, 3.19)	0.18 (0.04, 0.86) *	0.36 (0.18, 0.70) **	1
**Living With Comorbidities**						
No	Ref		Ref		Ref	
Yes	0.94 (0.63, 1.47)		0.99 (0.58, 1.70)		1.35 (0.91, 1.98)	
**Comorbidities**						
Arthritis	1		1			
Bodily pains	1		1			
Diabetes	2.43 (0.92, 6.43)		3.19 (0.86, 1.82)			
Diabetes &Hypertension	1.62 (0.38, 6.82)		1			
Hypertension	1		1			
Hernia	1		1			
Peptic ulcer	1		1			
Rheumatism	1		1			
Stroke	1		1			
**Are you currently on any behavioural/lifestyle treatment?**						
No	Ref		Ref		Ref	
Yes	1		1		1	
**Recommended behavioural/lifestyle changes**						
**Dietary Changes**						
No	Ref		Ref		Ref	
Yes	0.60 (0.35, 1.02)		2.26 (0.87, 5.89)		1	
**Physical Activity**						
No	Ref		Ref		Ref	
Yes	0.43 (0.20, 0.90) **	0.23 (0.09, 0.59) **	1		0.31 (0.03, 2.85)	
**Smoke Cessation**						
No	Ref		Ref		Ref	
Yes	1		1		1	
**Alcohol Intake Moderation**						
No	Ref		Ref		Ref	
Yes	0.92 (0.54, 1.57)		0.72 (0.32, 1.58)		1	

* p<0.05

**p<0.01

***p<0.001

### Influence of perceived social support on treatment adherence

Table 3 presents the summaries of the logistic regression analysis run to ascertain the influence of PSS on the treatment adherence of respondents. The results showed no significant influence of PSS on the three dimensions of treatment adherence. However, the PSS “Friends” subscale showed some level of significance with the lifestyle/behavioural dimension of adherence where respondents who reported low perceived support from friends (aOR = 8.58, 95% CI = 4.21, 17.52) were more likely to be adherent to their lifestyle/behavioural recommendations.

**Table 3:** Influence of perceived social support on treatment adherence.

Variables	Medication Adherence		Behavioural/Lifestyle Adherence		Review Adherence	
	cOR [95% CI]	aOR [95% CI]	cOR [95% CI]	aOR [95% CI]	cOR [95% CI]	aOR [95% CI]
**Perceived Social Support**						
High perceived support	Ref		Ref	Ref	Ref	
Low perceived support	1.17 (0.78, 1.74)		2.48 (1.46, 4.22) ***	0.74 (0.37, 1.49)	0.42 (0.05, 3.75)	
**Perceived support from significant other**						
High perceived support	Ref		Ref		Ref	
Low perceived support	1.10 (0.74, 1.63)		0.66 (0.39, 1.12)		0.21 (0.02, 1.87)	
**Perceived support from friends**						
High perceived support	Ref		Ref	Ref	Ref	
Low perceived support	1.43 (0.95, 2.18)		7.20 (4.06, 12.77) ***	8.58 (4.21, 17.52) ***	0.58 (0.06, 5.26)	
**Perceived support from family**						
High perceived support	Ref		Ref		Ref	
Low perceived support	1.19 (0.80, 1.77)		1.34 (0.79, 2.27)		0.38 (0.04, 3.42)	

* p<0.05

**p<0.01

***p<0.001

## Discussion

This study investigated the influence of perceived social support on treatment adherence in persons with chronic noncommunicable diseases who visited Ghana’s Ho Teaching Hospital. We found that respondents reported high levels of perceived social support (62%), medication adherence (62%), lifestyle/behavioural adherence (84.6%), as well as review adherence (98.8%). However, the highest level of non-adherence was recorded with respondents’ medication. Our study also found that respondents’ religion, ethnicity, the specific CNCD they had been diagnosed with, and the duration they have been living with the diagnosis are factors that influenced their level of treatment adherence. Concerning the influence of perceived social support on treatment adherence, we found that generalized perceived social support has no particular influence on respondents’ treatment adherence. However, we found that respondents who reported low perceived social support from friends were more likely to be adherent to the behavioural/lifestyle modification aspect of their treatment. Although our study reports high levels of perceived social support and treatment adherence, the level of non-adherence to medication found remains significant and could have negative implications for the general well-being of adults living with CNCDs as well as the achievement of the Sustainable Development Goals (SDGs), specifically target 3.4, which aims to reduce premature mortality from non-communicable diseases by one-third by 2030.

The results of the present study showed that more than half (62.04%) of the respondents were adherent to their medication. The medication adherence rate found in our study was significantly higher than that found in a study by Addo et al. [34] in which they posited a medication adherence rate of 45% in patients with chronic diseases attending a primary health facility in a peri-urban district in Ghana. However, our findings were consistent with those found by Awuni [35] who posited a medication adherence rate of 68.5% among persons living diabetes in a municipality in Ghana. Comparatively, our study results showed some level of consistency with indicated adherence rate in developed countries. WHO reports that “in developed countries, adherence among patients suffering chronic diseases averages 50 percent” [36]. Compared with previous international studies, the adherence rate assessed in this study was similar to, albeit slightly higher than the 53% found in Chinese primary-care centres [37], the 48% reported in uninsured American patients who attended community health centres [14], but slightly higher than the 39% observed in Italian outpatient adults [38]. At the national level, our findings are consistent with prior research in Spain performed on chronic disease patients [39], but significantly different in terms of the adherence rate of 18% reported in tertiary-care settings [40]. These variations in medication adherence have been indicated to be multifactorial. Some factors that could account for such variations as explored by other studies include lack of knowledge, financial problems, lack of family support, poor communication with healthcare providers, remote healthcare facility and scarcity of drugs [41, 42].

The results, similarly, indicated that most of the respondents remained adherent to their behavioural/lifestyle treatment (84.58%) as a significantly high proportion (98.83%) were adherent to their review appointments.

Additionally, the current study recorded age, ethnicity, and duration of diagnosis to be predictive of medication adherence among persons living with CNCDs. It was similarly reported that age, education, the CNCD diagnosis and the duration of the diagnosis served as predictors of adherence. However, the study showed no significant association between demographic baselines and review adherence among persons living with CNCDs.

Treatment adherence is a crucial aspect of chronic disease management, and understanding its predictors is essential for effective healthcare delivery. According to the current study, several factors influenced medication adherence, including religion, ethnicity, diagnosed CNCDs, diagnosis duration, and physical activity recommendation. Individuals who practice Islam were more likely to adhere to medication compared to Christians, while those of Guan and Ga/Dagme ethnicities were more likely to adhere than Akans. This was congruent to the findings of Wahab et al. [43] and Asiri et al. [44] which indicated ethnic and religious influence on medication adherence among persons living with diabetes and those living with hypertension, respectively. These underscore the continuous influence of ethno-religious factors on medication adherence [44]. This suggests the need for further studies to investigate the factors influencing treatment adherence across religious and ethnic groups within the Ghanaian society.

Additionally, patients diagnosed for longer durations had a lower likelihood of adherence, with the highest odds of adherence seen in patients diagnosed for less than a year. This finding is similar to that of Al-Noumani et al. [45], whose study indicated that patients diagnosed for more than 10 years exhibited lower adherence rates. This relationship has been corroborated by other studies, which suggest that the longer an individual lives with their diagnosis, the more likely they are to exhibit lower adherence rates [46]. Similarly, prolonged duration of diagnosis is posited to sometimes lead to depression, further contributing to reduced treatment adherence [47]. This underscores the importance of health literacy, effective communication with healthcare professionals, and robust social support systems, as these factors are known to positively influence treatment adherence [45].

Contrary to our hypothesis, perceived social support had no significant influence on respondents’ medication adherence behaviour. Similarly, none of the three subscales predicted medication adherence among our respondents. This finding conflicts with existing literature on perceived social support and medication adherence such as the study by Affusim and Francis [17] which found a significant association between social support and medication. Another study which postulated the influence of perceived social support on medication was by Asilar et al. [48]. However, the present study’s finding was consistent in parts with that of Anakwa et al. [49] who assessed the influence of perceived social support on medication adherence among persons living with HIV in Ghana.

The study found that individuals who perceived low social support from friends had higher adherence to behavioural/lifestyle treatments, including dietary changes, physical activity, and alcohol moderation or cessation, compared to those with high social support from friends. This finding was incongruent with that of Aschbrenner’s study [50], which highlighted the impact of family criticism on individuals’ readiness to change physical activity. This phenomenon could be explained by the perceived negative influence from friends as well as the family-oriented society such as that found in the Ho Municipality of Ghana. Additionally, this may be due to individuals’ increasing independence and responsibility for their well-being, reflecting the changing family and friends dynamics in diverse cultural contexts [51].

## Conclusion

This study investigated the levels of PSS and its influence on treatment adherence among adults with CNCDs. We found high levels of perceived social support and adherence to medication, behavioural/lifestyle recommendations and review appointments among respondents. However, the results of this study posit no influence of PSS on the three dimensions of treatment adherence investigated in the exception of individuals with low PSS from friends being more likely to be adherent to behavioural/lifestyle recommendations made by physicians. The high treatment adherence rate recorded in this study could be attributed to the participants’ capacity to access and engage health facilities. This implies the need for interventions aimed at reducing factors that may inhibit an individual’s ability to interact with health facilities in managing their disease conditions.

Additionally, the significant proportion of non-adherence to medication found among respondents is concerning as this may have possible negative effects on treatment outcomes in individuals with CNCDs, including the exacerbation of their conditions, thus perpetuating the elevated morbidity and mortality associated with CNCDs in Ghana. Moreover, these phenomena could potentially undercut interventions aimed at reducing CNCD-related mortality and morbidity, as well as hinder Ghana’s progress towards achieving target 3.4 of the Sustainable Development Goals (SDGs), which aims to reduce premature mortality from NCDs by one-third through prevention and treatment by 2030.

### Strengths and limitations

In this study, we provided important insights into the perceived social support and treatment adherence behaviour of adults living with CNCDs with particular interest in adherence to behavioural/lifestyle modification and review/checkup. The use of logistic regression ensured that we robustly established the relationships that exist between our outcome and explanatory variables. However, a limitation of the study is that we relied on self-reported data, which may be subject to recall bias and social desirability bias. Despite these limitations, the study can provide valuable information for healthcare providers and policymakers to help improve the lives of those living with chronic non-communicable diseases.

## Supporting information

S1 Checklist Reporting checklist. (DOCX)

S1 Data Collection instruments. (DOCX)

S1 Table Measurement of explanatory variables. (DOCX)
