# Supplementary material for: Influence of perceived social support and other factors on treatment adherence among adults living with chronic non-communicable diseases in the Ho Municipality of Ghana: A health facility-based cross-sectional study
Source: PLoS One. 2024 Sep 6;19(9):e0308402. doi: 10.1371/journal.pone.0308402 (PMC11379372; doi:10.1371/journal.pone.0308402)
Supplement: S1 Data — (DOCX) [file pone.0308402.s002.docx]

## Supplementary File 2 – Data Collection Instruments

**DATA COLLECTION INSTRUMENT I (QUESTIONNAIRE)**

**UNIVERSITY OF HEALTH AND ALLIED SCIENCES, HO**

**F. N. BINKA SCHOOL OF PUBLIC HEALTH**

This questionnaire is designed to assess the level of psychological distress, perceived social support and health-related quality of life among chronic non-communicable disease patients in the Ho Municipality. All information given on this questionnaire will be held confidential and used only for the purpose of the study.

| **For Official use only** | | | |
| --- | --- | --- | --- |
| QUESTIONNAIRE NO.: _______________ | | DATE OF INTERVIEW: ___/___/______ | |
|  |  |  |  |
| NAME OF INTERVIEWER: _______________________________ | | |  |

**Section 1: SOCIO-DEMOGRAPHIC CHARACTERISTICS**

| **Code** | **Variables** | **Responses** |  |
| --- | --- | --- | --- |
| Age | What is your age? (In completed years) | _______ |  |
| Sex | Sex of respondent? | 1. Male 2. Female | [ ]  [ ] |
| MarStat | What is your marital status? | 1. Never Married 2. Married 3. Divorced/Separated 4. Widowed | [ ]  [ ]  [ ]  [ ] |
| Edu_Level | What is your highest Educational Level? | 1. No Formal Education 2. Primary 3. JHS/JSS/Middle 4. SHS/SSS/O-Level 5. Tertiary | [ ]  [ ]  [ ]  [ ]  [ ] |
| Religion | What is your religion? | 1. Christianity 2. Islam 3. African Traditional 4. Other (specify) __________ | [ ]  [ ]  [ ]  [ ] |
| Ethnicity | What is your ethnicity? | 1. Akan 2. Ewe 3. Guan 4. Ga/Dangme 5. Mole-Dagbani 6. Other (specify) | [ ]  [ ]  [ ]  [ ]  [ ]  [ ] |
| Diagnosed CNCD | What chronic noncommunicable disease have you been diagnosed with? | ___________________ |  |
| Duration | How long have you been diagnosed with this condition? | ___________________ |  |
| Comorbidity | Are you currently living with any comorbidity? | 1. No 2. Yes   If yes please specify ­­­­­­­­______________ | [ ]  [ ] |

**Section 2: Social Support System**

This section of the questionnaire adopts the **Multidimensional Scale of Perceived Social Support (MSPSS)** (Zimet et al., 1988), a 12-item scale, to measure perceived adequacy of social support from three sources: family, friends, & significant other; using a 5-point Likert scale (0 = strongly disagree, 5 = strongly agree).

We are interested in how you feel about the following statements. Read each statement carefully. Indicate how you feel about each statement.

Select “1” if you **Very Strongly Disagree** ; “2” if you **Strongly Disagree** ; “3” if you **Mildly Disagree** ; “4” if you are **Neutral** ; “5” if you **Mildly Agree** ; “6” if you **Strongly Agree** and “7” if you **Very Strongly Agree**

|  |  | **1** | **2** | **3** | **4** | **5** | **6** | **7** |
| --- | --- | --- | --- | --- | --- | --- | --- | --- |
| **SS001** | There is a special person who is around when I am in need |  |  |  |  |  |  |  |
| **SS002** | There is a special person with whom I can share my joys and sorrows. |  |  |  |  |  |  |  |
| **SS003** | My family really tries to help me |  |  |  |  |  |  |  |
| **SS004** | I get the emotional help and support I need from  my family. |  |  |  |  |  |  |  |
| **SS005** | I have a special person who is a real source of  comfort to me. |  |  |  |  |  |  |  |
| **SS006** | My friends really try to help me |  |  |  |  |  |  |  |
| **SS007** | I can count on my friends when things go wrong |  |  |  |  |  |  |  |
| **SS008** | I can talk about my problems with my family |  |  |  |  |  |  |  |
| **SS009** | I have friends with whom I can share my joys and sorrows. |  |  |  |  |  |  |  |
| **SS010** | There is a special person in my life who cares  about my feelings |  |  |  |  |  |  |  |
| **SS011** | My family is willing to help me make decisions |  |  |  |  |  |  |  |
| **SS012** | I can talk about my problems with my friends. |  |  |  |  |  |  |  |

**Section 3: Treatment Adherence Behaviour**

**Part 1: Medication Adherence**

This part of the questionnaire seeks to determine your adherence your medication regimen using the Medication Adherence Rating Scale (MARS)

Please respond to the statements in the questionnaire by selecting the response which best describes your behaviour towards your medication ***during the past week***

| Codes | Questions | Responses | |
| --- | --- | --- | --- |
|  |  | True | False |
| MAD01 | Do you ever forget to take your medication? | [ ] | [ ] |
| MAD02 | Are you careless at times about taking your medication? | [ ] | [ ] |
| MAD03 | When you feel better, do you sometimes stop taking your medication? | [ ] | [ ] |
| MAD04 | Sometimes if you feel worse when you take the medication, do you stop taking it? | [ ] | [ ] |
| MAD05 | I take my medication only when I am sick | [ ] | [ ] |
| MAD06 | It is unnatural for my mind and body to be controlled by medication | [ ] | [ ] |
| MAD07 | My thoughts are clearer on medication | [ ] | [ ] |
| MAD08 | By staying on medication, I can prevent getting sick. | [ ] | [ ] |
| MAD09 | I feel weird, like a ‘zombie’ on medication | [ ] | [ ] |
| MAD10 | Medication makes me feel tired and sluggish | [ ] | [ ] |

**Part 2: Lifestyle and Review Adherence**

Please respond to the statements in the questionnaire by selecting the response which best describes your behavioural/lifestyle and review adherence to treatment.

| **Codes** | **Questions** | **Responses** |  |
| --- | --- | --- | --- |
| LAD01 | Are you currently on any behavioral/lifestyle treatment? | 1. No 2. Yes | **[ ]**  **[ ]** |
| LAD02 | *If yes:*  Which behavioral / lifestyle changes were recommended for you?  **[Select as many that applies]** | 1. Dietary changes 2. Physical activity 3. Smoke cessation 4. Alcohol intake moderation 5. Other (Specify) | **[ ]**  **[ ]**  **[ ]**  **[ ]**  **[ ]** |
| LAD03 | How often do you adhere to these recommendation(s)? | 1. All the time 2. Most of the time 3. Sometimes 4. Rarely | **[ ]**  **[ ]**  **[ ]**  **[ ]** |
| LAD04 | How often do you go for your follow up / check-up / review? | 1. Weekly 2. Every two weeks 3. Monthly 4. Every two months 5. Every three months | **[ ]**  **[ ]**  **[ ]**  **[ ]**  **[ ]** |
| LAD05 | How often do you adhere to your scheduled appointments for your follow up / check-up / review? | 1. All the time 2. Most of the time 3. Sometimes 4. Rarely | **[ ]**  **[ ]**  **[ ]**  **[ ]** |
