# Supplementary material for: Influence of perceived social support and other factors on treatment adherence among adults living with chronic non-communicable diseases in the Ho Municipality of Ghana: A health facility-based cross-sectional study
Source: PLoS One. 2024 Sep 6;19(9):e0308402. doi: 10.1371/journal.pone.0308402 (PMC11379372; doi:10.1371/journal.pone.0308402)
Supplement: S1 Table — (DOCX) [file pone.0308402.s003.docx]

**Supplementary File 3**

**3.1 Measurement of Explanatory Variables**

| **Variable** | **Measurement** |
| --- | --- |
| Age | Self-reported age in years categorized as 30 – 39, 40 – 49. 50 – 59, and 60+. |
| Sex | Self-reported gender as male or female |
| Marital status | Self-reported marital status as never married, married, divorced/separated, or widowed |
| Education | Self-reported highest level of education completed as no formal education, primary, JHS/JSS/Middle School, SHS/SSS/O-Level or tertiary. |
| Religion | Self-reported religious affiliation as Christianity, Islam, or African Traditional Religion. |
| Ethnicity | Self-reported ethnic affiliation as Akan, Ewe, Guan or Ga-Dangme. |
| Diagnosed CNCD | Self-reported diagnosis of a chronic non-communicable disease as cancer, Chronic kidney disease, diabetes, hypertension or stroke. |
| Diagnosis duration | Self-reported length of time in years since diagnosis as <1, 1 – 5, 6 – 10, or 10+. |
| Comorbidity status | Self-reported presence or absence of other health conditions |
| Specific comorbidities | Self-reported list of other health conditions |
| Recommended behavioural/lifestyle changes | Self-reported behavioural/lifestyle changes recommended by physicians and other health professionals, including physical activity, dietary changes, smoke cessation, and alcohol intake moderation |

**3.2 Operational Definition of Variables**

| **Variables** | **Definitions** |
| --- | --- |
| Perceived Social Support | Self-reported degree of social support received was measured using the Multidimensional Scale for Perceived Social Support. |
| Significant other | Anyone the respondent shares a romantic, committed, and intimate relationship with. |
| Treatment adherence | Overall adherence to all components of respondents’ treatment regimen. This encompasses their medication, behavioural/lifestyle and review adherence. |
| Medication adherence | Self-reported adherence to prescribed medication for diagnosed CNCDs |
| Behavioural/Lifestyle adherence | Self-reported adherence to behavioural/lifestyle modifications recommended by physicians for treating diagnosed CNCDs |
| Review adherence | Self-reported adherence to review/checkup/appointment attendance as scheduled. |
